# Failure of lysosomal acidification and endomembrane network in neurodegeneration

**DOI:** 10.1038/s12276-025-01579-x

**Published:** 2025-11-18

**Authors:** Seo-Hyun Kim, Young-Sin Cho, Yong-Keun Jung

**Affiliations:** 1 Department of Neurology and Institute for Cell Engineering, Johns Hopkins University School of Medicine, Baltimore, MD USA; 2 School of Biological Sciences, Seoul National University, Seoul, Republic of Korea; 3 The Research Institute of Basic Sciences, Seoul National University, Seoul, Republic of Korea

**Keywords:** Lysosomes, Neurodegeneration

## Abstract

Lysosomes have emerged as central hubs in the regulation of the endomembrane system, extending beyond degradation to coordinate organelle communication. Central to this regulatory role is vacuolar-type H^+^-ATPase (V-ATPase), a proton pump that acidifies the lysosomal lumen to enable hydrolase activity and support proteostasis. In addition to its lysosomal functions, V-ATPase influences the physiology of other organelles, including the endoplasmic reticulum (ER), Golgi apparatus and mitochondria, through both direct and indirect mechanisms involving acidification-dependent processes, such as protein folding, vesicular trafficking and stress responses. V-ATPase dysfunction compromises interorganelle communication through multiple mechanisms, including impaired calcium and lipid exchange at contact sites, disrupted organelle positioning and defective autophagic and stress signaling. In neurodegenerative diseases, such as Alzheimer’s and Parkinson’s diseases, V-ATPase impairment contributes to lysosomal storage pathology, ER stress, Golgi fragmentation and mitochondrial dysfunction. ER–endolysosome tethering proteins and mitochondria–lysosome contacts are particularly sensitive to pH and trafficking defects. These disruptions result in a cascade of organelle dysfunction and contribute to disease progression. Here, in this Review, we highlight how V-ATPase governs both local lysosomal function and broader organelle network integrity, positioning it as a critical regulator of endomembrane homeostasis and a potential therapeutic target in neurodegenerative conditions.

## V-ATPase links lysosomes to organelle communication and health

Lysosomes are now known to play important roles in cell signaling and maintaining balance within cells. This Review explores how lysosomes interact with other cell parts and their role in diseases such as Alzheimer’s and Parkinson’s. The authors focus on a protein complex called V-ATPase, a condition necessary for breaking down cellular waste. They used various models, including mice and flies, to study how V-ATPase dysfunction affects lysosome function and contributes to neurodegenerative diseases. The research shows that, when V-ATPase does not work properly, it leads to problems in lysosome function, affecting other cell parts such as the endoplasmic reticulum and mitochondria. This disruption can lead to the buildup of harmful substances in cells, contributing to diseases. The authors suggest that targeting V-ATPase to restore lysosomal acidity could be a promising treatment strategy for neurodegenerative diseases.

This summary was initially drafted using artificial intelligence, then revised and fact-checked by the author.

## Introduction

Lysosomes were once regarded solely as terminal degradative organelles, but this view has evolved substantially with the recognition of their broader roles in intracellular signaling and the regulation of vesicle fusion and recycling^1–5^. Recent studies have established lysosomes as dynamic platforms that integrate cues from metabolic status, nutrient availability and stress conditions to coordinate cellular homeostasis^2–5^. Central to this regulatory function is their ability to control mTORC1 activity through amino-acid-dependent recruitment of signaling complexes to the lysosomal membrane^3,4,6^. In addition to mTORC1, the lysosomal surface coordinates other signaling pathways, including TFEB, AMPK and TRPML1, which modulate autophagy, lysosome biogenesis and metabolic adaptation^7–9^. These signaling modules are spatially organized at the lysosomal membrane to facilitate cellular adaptation to fluctuations in nutrient, energy, and stress conditions.

To maintain normal lysosomal function, the lysosomal lumen must be properly acidified, as this acidic environment is essential for the activation of hydrolytic enzymes and cargo degradation. This acidification is primarily driven by the V-ATPase, a multi-subunit proton pump that actively transports protons into the lysosomal lumen^10,11^. V-ATPase also plays critical roles in endolysosomal dynamics, including phagocytosis, endocytosis and autophagy, by regulating organelle acidification and membrane trafficking^12^. The essential role of V-ATPase in maintaining lysosomal function has been demonstrated across diverse model organisms. In *Caenorhabditis elegans*, mutations in V-ATPase B subunit (vha-12) lead to lysosomal alkalinization, accumulation of autophagic vacuoles and locomotor defects^13^. In *Drosophila*, loss of the neuron-specific V0a1-subunit (v100) causes progressive photoreceptor degeneration and impaired degradation capacity, while deletion of the assembly factor *Atp6ap2* induces synaptic dysfunction and vacuolar accumulation^14,15^. Zebrafish carrying mutations in *atp6v1e1b* or *atp6v0a3* exhibit developmental abnormalities, defective phagolysosome maturation in microglia and disrupted autophagic clearance^12,16^. In addition, conditional knockout models in mice show that neuron-specific deletion of *Atp6v0a1* (V0a1) or *Atp6ap2* results in hippocampal atrophy, autophagosome accumulation and synaptic failure^14,17^. These findings collectively support a conserved requirement for V-ATPase in neuronal homeostasis and lysosomal proteolysis.

In line with these genetic observations, V-ATPase dysfunction has also been implicated in neurodegenerative disease models. Loss of V0a1 subunit-dependent degradation has been implicated in Alzheimer’s disease (AD) models^18^. Similarly, mislocalization of the V0a1 subunit disrupts lysosomal acidification and leads to the lysosomal storage pathology in a genetic neurodegeneration model^19^. In neurons, the impairment of V-ATPase-dependent acidification leads to the accumulation of undegraded substrates, contributing to the pathogenesis of neurodegenerative diseases, such as AD and Parkinson’s disease (PD)^10^. Specifically, in AD, direct binding of amyloid-β (Aβ) or tau to V-ATPase impairs its acidifying function, further compromising endolysosomal integrity in AD^20^. Recent evidence from AD mouse models demonstrated that the reduced neuronal autolysosome acidification, driven by compromised lysosomal V-ATPase activity, precedes extracellular amyloid plaque formation and promotes intraneuronal accumulation of Aβ within enlarged autophagic structures termed PANTHOS, which subsequently develop into senile plaques^21^. Furthermore, V-ATPase activity influences lysosomal calcium (Ca^2+^) release and membrane trafficking events, linking proton transport with broader aspects of lysosomal homeostasis^10,22^. Lysosomal acidification, maintained by V-ATPase, is essential for the activity of lipid-processing enzymes involved in cholesterol and sphingolipid metabolism^22,23^. Disruption of this acidic environment impairs enzyme function and hinders cholesterol export, thereby linking V-ATPase activity with lysosomal lipid clearance^22,23^.

The lysosomal surface hosts numerous molecular interactions that connect it with other organelles, including the ER, the Golgi apparatus and mitochondria, thereby influencing trafficking routes and membrane remodeling processes^1,2,4,5^. These interorganelle communications are further modulated by the lysosome’s spatial positioning and pH-dependent enzyme activity, both of which are dynamically regulated in response to cellular demands^2–5^. To experimentally interrogate these processes, several tools have been developed to monitor V-ATPase-dependent lysosomal acidification. These include pH-sensitive fluorescent probes, such as LysoSensor dyes, ratiometric dextrans and genetically encoded lysosomal probes, enabling real-time and compartment-specific monitoring of luminal pH^24,25^. Mechanistic insights have also been gained through genetic disruption of V-ATPase subunits and pharmacological inhibition of V-ATPase^26,27^. These methods have been instrumental in validating the role of lysosomal acidification in regulating interorganelle communication and in assessing how its restoration may correct disease-associated defects in organelle dynamics.

Endoplasmic reticulum (ER)–lysosome contact sites regulate lipid and Ca^2+^ exchange, and these processes are influenced by V-ATPase-dependent lysosomal acidification^28–31^. Similarly, the Golgi relies on pH gradients maintained by V-ATPase for the proper sorting and delivery of lysosomal enzymes^32–35^, and mitochondria–lysosome contact sites (MLCs) coordinate mitophagy and energy homeostasis through pH- and Ca^2+^-dependent mechanisms^36–38^. Thus, V-ATPase-mediated acidification serves as a unifying mechanism linking lysosomal function with the maintenance of organelle network integrity. In neurodegenerative diseases, impaired lysosomal acidification disrupts autophagic clearance, mitochondrial quality control and interorganelle signaling, driving a progressive deterioration of endomembrane system integrity^39–41^. Until now, most information on the role of lysosome acidification in neurodegenerative diseases has been collected from neuronal cells.

In glial cells, impaired lysosomal acidification and disrupted lysosomal biogenesis, particularly in microglia, also contribute to proteostatic failure and heightened inflammatory responses, thereby exacerbating neurodegenerative progression^42,43^. In astrocytes, regulation of TFEB activity has been shown to influence lysosomal uptake and degradation of extracellular tau, potentially affecting its pathological spread^44^. In addition, mitochondrial dysfunction in glial cells has been linked to altered mitochondria–lysosome crosstalk, impairing lysosomal clearance capacity and further contributing to cellular stress responses associated with neurodegeneration^45^. Furthermore, ApoE4 expression has been associated with lysosomal dysfunction across multiple brain cell types, including astrocytes and neurons^46,47^. Specifically, ApoE4 impairs lysosomal cholesterol handling, endolysosomal trafficking and acidification, thus contributing to disrupted proteostasis and mitochondrial stress in AD models^46–48^. These findings suggest that targeting lysosomal acidification and restoring endomembrane homeostasis may represent an effective therapeutic strategy for neurodegeneration.

This Review highlights the multifaceted roles of lysosomes and V-ATPase in regulating degradation, signaling and organelle communication with an emphasis on how their dysfunction contributes to neurodegenerative disease pathology. By examining lysosome-centered interactions with the ER, Golgi apparatus and mitochondria, we aim to provide a comprehensive framework for understanding lysosomal regulation in health and disease.

## ER–endolysosome axis in endolysosomal dysfunction and stress response

### ER–endolysosome tethering for lipid transport and organelle positioning

**Fig. 1:**
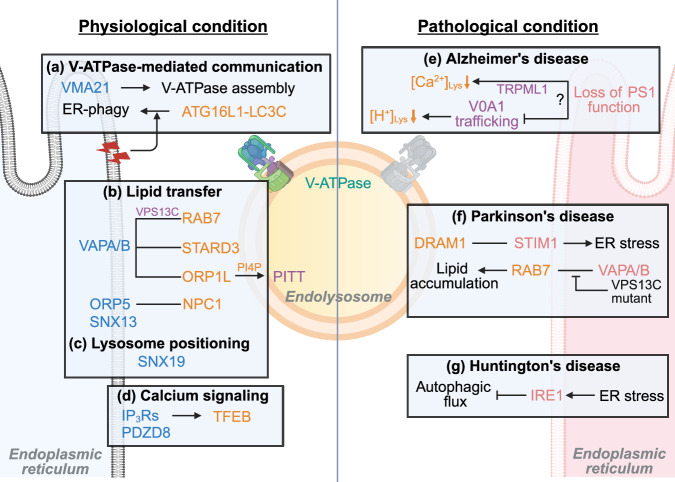
ER–endolysosome crosstalk under physiological and pathological conditions. **a**–**d** ER–endolysosome crosstalk under physiological conditions: V-ATPase V0 subunits are assembled in the ER with the aid of accessory proteins VMA21, ensuring proper complex maturation and trafficking. V-ATPase contributes ER proteostasis by mediating reticulophagy through the ATG16L1–LC3C axis, enabling selective clearance of damaged ER (**a**). VPS13C mediates direct lipid transport at ER–endolysosome interfaces by bridging ER-localized VAP-A/B and lysosomal Rab7. STARD3 anchors endolysosomes to the ER via VAPs. ORP5 promotes cholesterol transfer from endolysosomes to the ER, while ORP1L controls endolysosome positioning through VAPs. Upon lysosomal injury, the PITT pathway is activated; PI4K2A produces PI4P on the cytosolic surface of damaged lysosomes, facilitating the recruitment of ORP1L, OSBP and VAP-A/B to form ER–lysosome contact sites. SNX13 facilitates lysosomal cholesterol export (**b**) and SNX19 restricts endolysosome motility, supporting perinuclear retention (**c**). IP_3_Rs localize at ER–lysosome interfaces to mediate Ca^2+^ transfer from the ER to lysosomes, and IP_3_Rs-dependent Ca^2+^ release from the ER promotes TFEB activation. PDZD8 maintains contact site stability and assists lipid transfer, indirectly contributing to Ca^2+^ signaling by maintaining ER–endolysosome architecture (**d**). **e**–**g** ER–endolysosome crosstalk under pathological conditions: In AD, loss of PS1 function reduces lysosomal Ca^2+^ content and impairs acidification and autophagosome clearance. PS1 is also required for ER-to-lysosome trafficking of the V0a1 subunit, essential for V-ATPase assembly and lysosomal proteolysis (Controversy) (**e**). In PD, excess DRAM1 promotes ER–lysosome tethering through its interaction with STIM1, altering Ca^2+^ dynamics and aggravating ER stress. VPS13C, mutated in familial PD, localizes at ER–lysosome contact sites and its loss disrupts lipid exchange, leading to lysosomal accumulation of Di-22:6-BMP (**f**). In HD, activation of the ER stress sensor IRE1 impairs autophagic flux, leading to mHtt accumulation and disrupted lysosomal clearance (**g**).

ER–endolysosome contact sites are emerging as critical platforms for the regulation of lipid transfer and organelle positioning^29,49,50^ (Fig. 1). VPS13C, a lipid transport protein localized at the ER–lysosome interfaces, facilitates the direct movement of lipids between membranes, thereby contributing to the lysosomal membrane composition and function^29^. The molecular tethering between the ER and endolysosomes is mediated by STARD3 or its homolog STARD3NL, which interact with ER-resident VAP proteins to stabilize these contacts^49^. Oxysterol-binding protein-related proteins (ORPs), including ORP5 and ORP1L, also play key roles at ER–endolysosome contact sites. ORP5 regulates the transfer of cholesterol from endolysosomal membranes to the ER, thereby facilitating cholesterol trafficking^30^. Meanwhile, ORP1L interacts with VAP to control endolysosome positioning by modulating the Rab7–RILP–p150Glued complex, which links endolysosomes to the microtubule network^50^. In addition, ORP1L-mediated cholesterol efflux from endolysosomes further underscores its role in maintaining intracellular lipid balance^51^. Recent studies have expanded the list of ER–endolysosome tethering proteins to include SNX family members. SNX13, an ER-anchored sorting nexin, promotes cholesterol export from lysosomes, showing a functional role in lysosomal lipid clearance^52^. Similarly, SNX19 has been shown to restrict endolysosome motility by forming contacts with the ER, thereby promoting the retention of endolysosomes in the perinuclear region^53^.

ER–lysosome contact sites also mediate lipid-based membrane repair following lysosomal damage. A recently characterized pathway, phosphoinositide-initiated membrane tethering and lipid transport (PITT), is activated upon lysosomal injury^54^. In this pathway, PI4K2A generates PI4P at the cytosolic face of damaged lysosomes, which recruits ORP1L, OSBP and VAP-A/B to establish ER–lysosome contact sites. These tethering proteins promote the exchange of cholesterol and phosphatidylserine (PS) from the ER, stabilizing the compromised membrane. PS accumulation subsequently promotes the recruitment of large lipid transporters, such as ATG2 and VPS13C, enabling bulk membrane delivery from the ER^55^. Notably, VPS13C, a gene mutated in familial PD, functions as an early responder in this repair process, linking lysosomal recovery capacity to neurodegenerative vulnerability^56^. Collectively, these findings demonstrate that the ER–endolysosome contact site proteins orchestrate lipid trafficking and the spatial organization of the endolysosomal system, contributing to broader aspects of cellular homeostasis.

### ER–endolysosome contact for Ca^2+^ homeostasis

Emerging evidence indicates that ER–endolysosome contact sites serve as specialized platforms for the regulation of intracellular Ca^2+^ dynamics^28,57,58^ (Fig. 1). IP_3_ receptors (IP_3_Rs), a major class of ER-Ca^2+^ channels, have been shown to preferentially localize at ER–lysosome interfaces, where they facilitate the targeted delivery of Ca^2+^ from the ER to lysosomes^28^. This spatially restricted Ca^2+^ signaling is thought to contribute to lysosomal function and homeostasis^28^. IP_3_R-mediated Ca^2+^ release from the ER increases cytosolic Ca^2+^ concentrations, leading to calcineurin activation and subsequent dephosphorylation of TFEB/TFE3^59,60^. Similarly, resveratrol was shown to enhance lysosomal function by triggering ER-Ca^2+^ release and promoting TFEB nuclear import via a calcineurin-dependent mechanism^61^. Collectively, precise regulation of ER-derived Ca^2+^ flux at ER–lysosome contact sites is essential not only for maintaining lysosomal homeostasis under physiological conditions but also for orchestrating adaptive responses to cellular stress. Accordingly, dysregulation of this signaling may contribute to the pathogenesis of lysosome-related disorders.

As endosomes mature along the endocytic pathway, the extent of ER contact progressively increases, suggesting that dynamic ER–endolysosome interactions are involved in coordinating organelle identity and function during trafficking^57^. These contacts not only regulate Ca^2+^ transfer but also modulate the biophysical properties of endolysosomes, potentially affecting their positioning and maturation^57^. Structural proteins, such as PDZD8, have been implicated in maintaining the architecture of ER–endolysosome contacts, supporting lipid transfer and possibly contributing indirectly to Ca^2+^ signaling by stabilizing membrane interfaces^62^. Although PDZD8 is primarily associated with lipid transport, its role in maintaining contact site integrity underscores the multifaceted nature of the ER–endolysosome communication^62^. Finally, alterations in ER morphology and disruptions in ER–endolysosome interactions have been linked to impaired Ca^2+^ homeostasis and are emerging as pathogenic mechanisms in various neurodegenerative diseases^58^. Taken together, these findings indicate the importance of ER–endolysosome contact sites in regulating localized Ca^2+^ signaling, lipid transfer, insufficient membrane repair, organelle maturation and cellular homeostasis.

### Roles of V-ATPase in ER homeostasis and stress regulation

The assembly of V-ATPase complexes is critically dependent on the ER, where accessory proteins, such as ATP6AP2 and VMA21, facilitate the proper maturation and trafficking of V-ATPase subunits^4,40,63,64^. Disruption of these assembly factors impairs V-ATPase biogenesis, leading to defects in the acidification of downstream compartments and contributing to ER stress^64^ (Fig. 1). In addition, recent findings have revealed a direct link between V-ATPase activity and reticulophagy, a specialized form of autophagy that regulates ER quality^65^. Specifically, the V-ATPase–ATG16L1–LC3C axis mediates the selective clearance of damaged ER, showing a mechanism by which V-ATPase supports ER proteostasis^65^. Beyond its assembly and reticulophagic functions, V-ATPase-dependent lysosomal acidification plays an indirect but critical role in maintaining ER homeostasis. Impaired lysosomal function resulting from defective acidification can activate a lysosomal stress response, which triggers compensatory signaling pathways, including activation of transcription factors TFEB and TFE3^60,66^. Activation of these pathways promotes lysosomal biogenesis and autophagic flux, but prolonged or unresolved lysosomal stress can propagate ER stress, linking lysosomal dysfunction with broader disruptions in cellular homeostasis^60,66^. These findings illustrate the multifaceted roles of V-ATPase in regulating ER integrity, through both its direct assembly-dependent processes and its indirect modulation via lysosomal acidification and stress signaling.

### ER–lysosome crosstalk in neurodegenerative diseases

The interplay between the ER and lysosomes has become a determinant of neuronal health, particularly in the context of AD^41^ (Fig. 1). ER–lysosome contact sites coordinate Ca^2+^ exchange and lysosomal function, and their disruption is increasingly recognized as a contributing factor in AD pathogenesis^41^. In particular, presenilin 1 (PS1), best known for its role in γ-secretase activity, has been implicated in the regulation of lysosomal Ca^2+^ and proton homeostasis^31^. A study suggested that PS1 is required for the ER-to-lysosome trafficking of the V0a1 subunit of V-ATPase, and that its loss impairs lysosomal acidification and proteolysis, contributing to autophagic dysfunction in AD models^67^. However, subsequent reports challenged this model, suggesting that lysosomal Ca^2+^ dysregulation, rather than impaired proton pump activity, could contribute more substantially to the endolysosomal dysfunction in PS1-deficient cells^68,69^. Further investigation revealed that PS1 influences lysosomal Ca^2+^ homeostasis by modulating TRPML1 activity and that this regulation may involve V-ATPase-dependent acidification^31^. These findings reflect an ongoing debate as to whether lysosomal defects associated with PS1 deficiency are more closely linked to disrupted proton gradients, impaired Ca^2+^ signaling or a combination of both processes. These defects in lysosomal homeostasis could propagate stress responses that can perturb ER function, suggesting a bidirectional relationship between ER and lysosome dysfunction in AD^41^. Notably, altered ER–lysosome Ca^2+^ signaling in PS1-deficient models reflects a broader failure in the interorganelle communication, implicating these contact sites as key pathological nodes in AD-related neurodegeneration^41^.

Disruptions in ER–lysosome communication have also been implicated in the pathogenesis of PD. In fibroblasts derived from patients with GBA1-linked PD, remodeling of ER and lysosomal Ca^2+^ stores has been observed, indicating that defects in the interorganelle Ca^2+^ handling may contribute to disease progression^70^. Altered Ca^2+^ dynamics between the ER and lysosomes can impair lysosomal function, leading to the accumulation of undegraded substrates and exacerbation of cellular stress responses^70^. Mechanistically, proteins regulating membrane fusion and trafficking, such as the SNARE protein YKT6, are critical for maintaining organelle integrity under stress conditions^71^. Disruption of YKT6-mediated pathways by pathological α-Synuclein (α-Syn) accumulation compromises vesicular transport and may indirectly affect ER–lysosome interactions by impairing autophagic clearance mechanisms^71^. Recent findings further show that proteins directly promoting ER–lysosome contact formation are vital for Ca^2+^ and stress regulation in PD models. DRAM1, a p53 target gene, has also been shown to facilitate the ER–lysosome tethering via STIM1, thereby modulating Ca^2+^ homeostasis and exacerbating ER stress^72^. Moreover, lipid transfer protein VPS13C, mutated in familial PD, localizes at ER–lysosome interfaces and prevents aberrant STING signaling, illustrating a broader role of ER–lysosome crosstalk in regulating inflammatory and stress responses^73^ (Fig. 1).

The impaired ER–lysosome communication has been implicated in several neurodegenerative disorders beyond AD and PD. In Niemann–Pick disease type C, defective cholesterol trafficking results in abnormal regulation of ER–lysosome contact sites, leading to aberrant mTORC1 activation and dysregulated cellular growth signaling^74^. This finding shows how disrupted lipid sensing at ER–lysosome interfaces can contribute to disease pathogenesis^74^. In hereditary spastic paraplegia, mutations affecting ER–endosome tethering proteins impair lysosome function by disrupting the maturation and trafficking of endolysosomal compartments^75^. The failure to maintain appropriate ER–endolysosome contacts compromises lysosomal positioning and degradative capacity, contributing to axonal degeneration^75,76^. Similarly, ER–lysosome contacts at pre-axonal regions have been shown to regulate lysosome availability in axon, suggesting that defects in this process exacerbate neurodegenerative progression in motor neuron diseases^76^. In Huntington’s disease (HD) models, ER stress responses are closely linked to lysosomal dysfunction. Activation of the UPR sensor IRE1 impairs autophagic flux, promoting the accumulation of mutant huntingtin (mHtt) aggregates and further disturbing lysosomal clearance mechanisms^77^ (Fig. 1). Although the direct involvement of ER–lysosome contacts requires further elucidation, these findings emphasize the interplay between ER function, autophagy and lysosomal homeostasis in neurodegeneration^77^. Overall, the impairments in ER–lysosome communication, including defects in Ca^2+^ handling, lipid metabolism and lysosomal positioning, have been observed across AD, PD and a range of other neurodegenerative conditions, suggesting a broader relevance of the interorganelle signaling to disease pathophysiology.

## Golgi–lysosome crosstalk: defects of lysosomal acidification and trafficking in neurodegeneration

### Golgi as a trafficking hub for lysosomal proteins and lipids

**Fig. 2:**
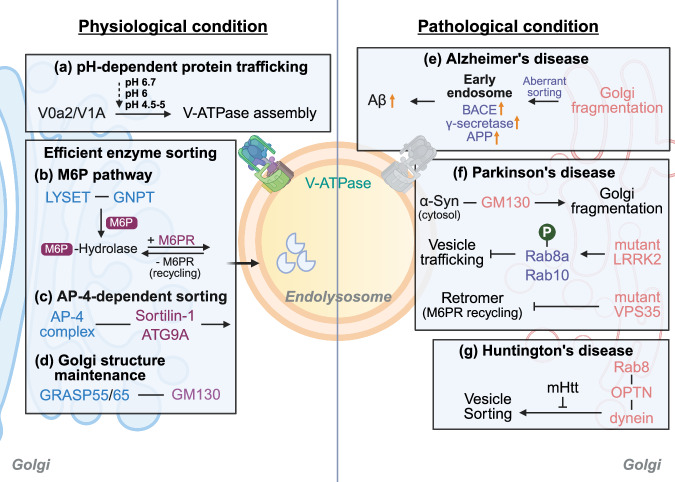
Regulation of Golgi-to-lysosome trafficking under physiological and pathological conditions. **a**–**d** Golgi-to-lysosome trafficking under physiological conditions: In healthy cells, a Golgi-to-lysosome pH gradient (Golgi ~6.7 to lysosomes ~4.5-5) maintained by V-ATPase (subunits V0a2 and V1A) ensures proper sequential binding and release of lysosomal enzymes and their receptors, facilitating precise cargo sorting (**a**). Lysosomal enzymes are targeted via the M6P pathway, which depends on the trafficking factor LYSET for proper M6P tagging and recognition. Lysosomal pro-hydrolases are tagged with M6P and bound by M6PR in the Golgi, ensuring their delivery to endosomes/lysosomes (**b**). The AP-4 adaptor complex mediates clathrin-dependent trafficking of sorting-specific cargos, such as Sortilin-1 and ATG9A, from the TGN to endolysosomes, thus maintaining lysosomal membrane integrity and neuronal autophagy homeostasis (**c**). Golgi structural proteins GRASP55/65 together with their multimerization partner GM130 preserve Golgi ribbon integrity, which is crucial for efficient enzyme sorting and vesicle formation (**d**). **e**–**g** Golgi-to-lysosome trafficking under pathological conditions: In AD, early Golgi fragmentation disrupts sorting, causing aberrant trafficking of amyloid-processing proteins (BACE1, γ-secretase and APP) to early endosomes, where their colocalization drives excessive APP cleavage and pathological Aβ generation (**e**). In PD, cytosolic α-Syn abnormally associates with the Golgi matrix protein GM130, inducing Golgi structural disorganization and fragmentation in dopaminergic neurons. Concurrently, familial PD mutations in LRRK2 result in aberrant phosphorylation of Rab8a and Rab10, which impairs their normal function in vesicle trafficking and cargo sorting at the Golgi. Another PD-linked defect involves the retromer complex; mutant VPS35 fails to efficiently mediate retrograde transport of receptors from endosomes back to the Golgi. This includes impaired retrieval of the M6P receptor and other cargo, leading to deficits in lysosomal enzyme recycling and exacerbating lysosomal dysfunction (**f**). Wild-type (WT) Htt normally scaffolds a trafficking complex with Rab8 and OPTN, which links Golgi-derived vesicles to the dynein motor for delivery to degradative compartments. In HD, mHtt disrupts this complex, impairing vesicle sorting and lysosomal trafficking and causing autophagosome misrouting, protein aggregate accumulation and defective degradation (**g**).

The Golgi apparatus, centrally positioned in the secretory pathway, acts as a trafficking hub for the delivery of synthesized proteins and lipids to lysosomes^35,78^. Newly synthesized lysosomal hydrolases and membrane proteins transit through the Golgi and are sorted into appropriate vesicles within the trans-Golgi network (TGN) for delivery to endosomes and lysosomes^32,79^ (Fig. 2). In addition, the Golgi apparatus supplies lipids essential for lysosomal membrane integrity via vesicular transport and organelle contact sites, thereby maintaining lysosomal membrane composition^80^. Efficient transport of proteins and lipids through the Golgi apparatus is thus important for ensuring lysosomal enzyme activity and membrane integrity^81,82^.

Transport of lysosomal enzymes between the Golgi and lysosomes predominantly utilizes the mannose-6-phosphate (M6P) pathway^83^. Hydrolases synthesized in the ER acquire an M6P tag within the TGN, serving as a recognition signal for binding to M6P receptors (M6PR) and facilitating their targeted delivery to lysosomes^83^. Recent studies identified LYSET/TMEM251 (Golgi complex-associated factor, GCAF) as a critical regulator of this process^84^. LYSET/TMEM251 stabilizes the Golgi-resident GlcNAc-1-phosphotransferase (GNPT) complex for appending M6P tags to hydrolases, thus ensuring precise enzyme delivery to lysosomes. Dysfunctional LYSET/TMEM251 prevents M6P tagging, leading to secretion of extracellular enzymes and substantial impairment of lysosomal hydrolase activity^84^. After delivery, M6PRs are recycled back to the TGN by the retromer complex or Golgi-associated Rab GTPases, such as Rab9^85^. This Golgi–endosome–lysosome crosstalk is bidirectional^82^. The Golgi apparatus facilitates lysosomal biogenesis by supplying enzymes and membrane proteins, while lysosomes and endosomes return receptors and membrane components to the Golgi for ongoing sorting processes. Clathrin-coated vesicle formation and adaptor protein complexes (AP-1, AP-3 and AP-4) regulate this trafficking, directing cargo-laden vesicles from the TGN to lysosomes^82^. For instance, the AP-4 complex sorts lysosomal receptors, such as sortilin-1, from the TGN to the lysosomal pathway, essential for functional lysosome generation in neurons^86^.

Functional interplay of the Golgi–lysosome axis is modulated by various contact sites and signaling pathways. Golgi matrix proteins GM130 and GRASP55/65 cooperatively stabilize Golgi cisternal stacking and ribbon integrity, which is crucial for efficient vesicular trafficking and accurate protein sorting^83^. Loss or dysfunction of GRASP proteins disrupts enzyme mannose phosphorylation, causing aberrant extracellular secretion of enzyme precursors and lysosomal enzyme depletion, thus impairing lysosomal function^83^. Consequently, Golgi dysfunction can lead to enzyme missorting and extracellular release, notably decreasing lysosomal degradation capacity. In addition, anomalies in Golgi-associated factors, such as tethering proteins and vesicle-forming proteins, may disrupt lysosomal membrane protein composition and autophagosome–endosome fusion, broadly impacting cellular degradation pathways^81,86–88^.

### Role of V-ATPase in golgi function

The Golgi apparatus and lysosomes share mechanisms for pH regulation predominantly through V-ATPase, which maintains distinct luminal pH values (~6 in the Golgi and ~4.5–5 in lysosomes) and is critical for protein sorting and enzymatic activities^34^ (Fig. 2). V-ATPase dysfunction, such as loss of the V0a2 subunit, elevates Golgi and TGN pH, disrupting Golgi glycosyltransferase function and causing widespread glycosylation defects as well as delayed anterograde transport through the secretory pathway^89^. For example, the M6P receptor binds to hydrolases in the mildly acidic Golgi environment and releases them in the more acidic late endosomes^34^. Dysfunction in this mechanism leads to impaired hydrolase delivery, leaving many enzymes inactive. In addition, models lacking the V1 subunit A1 of V-ATPase exhibit impaired anterograde trafficking of membrane proteins, such as rhodopsin from the Golgi^90^. Similarly, loss of V-ATPase subunit function in plant cells disrupts storage protein trafficking beyond the Golgi, underscoring its evolutionarily conserved role in eukaryotes^33^. Experiments using V-ATPase inhibitors demonstrate structural changes and swelling of the Golgi, interfering with proper lysosomal enzyme transport^33^. Collectively, functional defects of V-ATPase compromise Golgi–lysosome-mediated hydrolase delivery and activation, potentially disrupting intracellular proteostasis.

### Golgi–lysosome crosstalk in neurodegenerative disease

Molecular crosstalk between the Golgi apparatus and lysosomes is critical in the specialized cells, such as neurons, where disruption in any single component may trigger dysfunction across interconnected organelles, leading to neurodegeneration. Indeed, impairment in the Golgi–lysosome interactions is commonly observed in various neurodegenerative diseases (Fig. 2). In AD, structural damage to the Golgi apparatus and lysosomal dysfunction appear as early pathological events^91^. Neurons from patients with AD characteristically exhibit Golgi fragmentation, preceding tau hyperphosphorylation and potentially exacerbating tau pathology^91,92^. Moreover, Aβ accumulation is closely associated with Golgi dysfunction. Proper APP processing and delivery to lysosomes rely on intact Golgi function; thus, Golgi fragmentation causes the missorting of APP-processing enzymes, elevating Aβ production and aggravating AD pathology^93^. Recent studies illustrated the role of AP-4 adaptor complex in regulating Golgi-to-lysosome trafficking of enzymes and membrane proteins. AP-4 deficiencies result in abnormal accumulation of APP-processing enzymes, exacerbating Aβ pathology^94^.

In PD, α-Syn disrupts Golgi structure by binding Golgi matrix proteins, such as GM130, impairing normal enzyme trafficking to lysosomes^95^. Dysfunction of Rab GTPases such as Rab1a, critical for forming and trafficking enzyme-containing vesicles from the Golgi, reduces lysosomal enzyme availability, diminishing degradation capacity and promoting the accumulation of pathological α-Syn^95^. Mutations in the *LRRK2* gene, common in PD, regulate Golgi–lysosome vesicular transport by aberrantly phosphorylating Rab proteins (Rab8a and Rab10), disrupting enzyme and membrane protein delivery to lysosomes and impairing proteostasis^96^. In addition, mutations in retromer components such as VPS35 disrupt receptor recycling between the Golgi and endosomes, causing lysosomal enzyme mislocalization^97^.

In HD, the accumulation of mHtt disrupts Golgi–lysosome trafficking. Normal huntingtin forms complexes with optineurin (OPTN) and Rab8, facilitating vesicle transport from the TGN. The mHtt disrupts this complex, misdirecting enzymes and membrane proteins intended for lysosomes, leading to toxic protein accumulation^98^. Amyotrophic lateral sclerosis (ALS) and frontotemporal dementia share genetic disruptions (for example, C9orf72, OPTN, VCP, TMEM106B and progranulin (PGRN)) that impair normal Golgi–endolysosomal trafficking, severely reducing intracellular protein handling and degradation capabilities^99^. C9orf72 mutations are known to disrupt Golgi-to-endosome receptor recycling, resulting in abnormal endosomal and lysosomal accumulation. In addition, dipeptide-repeat proteins mutations in OPTN, VCP, TMEM106B and PGRN similarly impair vesicular transport and lysosomal acidification, leading to intracellular accumulation of abnormal organelles and pathological proteins, including TDP-43^99^.

## Mitochondria–lysosome interplay in V-ATPase-mediated signaling and metabolism

### Functional cooperation between mitochondria and lysosomes

**Fig. 3:**
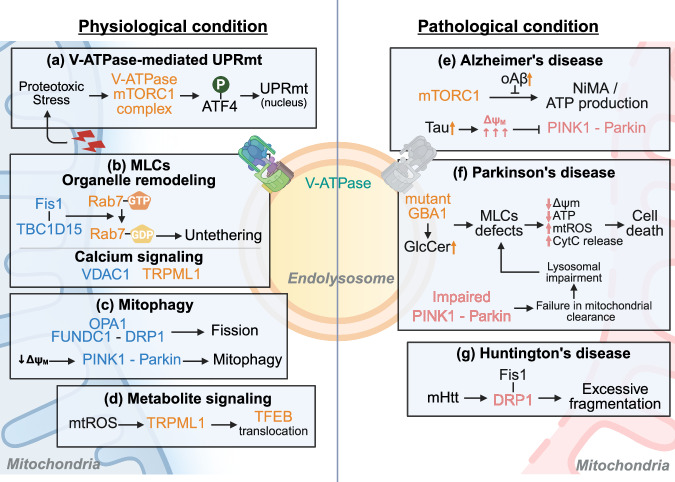
Mitochondria–lysosomal communications in MLCs under physiological and pathological conditions. **a**–**d** Mitochondria–lysosomal communications under physiological conditions: under mitochondrial proteotoxic stress, lysosomal v-ATPase–mTORC1 signaling is activated and directly phosphorylates the transcription factor ATF4, inducing UPRmt (**a**). Mitochondria and lysosomes form MLCs to coordinate organelle remodeling and metabolite exchange. At these contacts, the outer mitochondrial membrane protein Fis1 recruits the Rab7 GTPase-activating protein TBC1D15, driving Rab7-GTP hydrolysis and untethering the two organelles. Concurrently, lysosomal Ca^2+^ released via the TRPML1 channel is taken up by mitochondria through VDAC1 channels at MLC interfaces, which stimulates mitochondrial metabolic activity (**b**). Mitochondrial quality control is maintained through mitophagy. This involves several signaling pathways including the OPA1–FUNDC1–DRP1 axis, wherein stress-induced dissociation of the mitophagy receptor FUNDC1 from OPA1 facilitates DRP1-mediated mitochondrial fission and subsequent autophagosome recruitment. Another prominent pathway involves PINK1 and Parkin, where a mild depolarization of mitochondrial membrane potential (Δ*ψ*_m_) stabilizes PINK1 on the outer mitochondrial membrane, initiating Parkin recruitment and subsequent ubiquitin-dependent mitophagy through adaptor proteins such as OPTN (**c**). Mitochondria and lysosomes interact together via metabolite-mediated signaling mechanisms, including mtROS-TRPML1 activation, which leads to the TFEB-mediated lysosomal biogenesis, and NAD^+^ depletion, which causes V-ATPase dysfunction and lysosomal impairment (**d**). **e**–**g** Mitochondria–lysosomal communications under pathological conditions. In healthy cells, growth signals activate lysosomal mTORC1 to stimulate nutrient-induced mitochondrial activation (NiMA), enhancing mitochondrial respiration and ATP production. In AD, oligomeric Aβ disrupts this lysosome-to-mitochondria signaling to abrogate NiMA. Meanwhile, tau pathology induces an acute hyperpolarization of the Δ*ψ*_m_, which prevents PINK1 accumulation on mitochondria and thereby impairs the PINK1–Parkin-mediated mitophagy (**e**). In PD, loss-of-function mutations in GBA1 lead to glucosylceramide (GlcCer) accumulation in lysosomes, disrupting lysosomal integrity and impairing MLCs dynamics. The ensuing failure of metabolite exchange and organelle crosstalk leads to mitochondrial dysfunction, characterized by a reduced Δ*ψ*_m_ and ATP synthesis along with elevated ROS and cytochrome c release, ultimately triggering cell death. In addition, defects in the PINK1–Parkin pathway substantially impair selective mitochondrial clearance, exacerbating mitochondrial dysfunction and secondarily triggering lysosomal impairment, thus reinforcing a pathological cycle (**f**). DRP1 partners with Fis1 to execute mitochondrial fission. In HD, mHtt aberrantly interacts with and hyperactivates DRP1, driving excessive mitochondrial fragmentation. The overabundance of fragmented mitochondria overwhelms the autophagic-lysosomal degradation capacity, contributing to neuronal dysfunction and degeneration (**g**).

Mitochondria and lysosomes function cooperatively in metabolism and signaling to maintain cellular homeostasis^37^ (Fig. 3). Proper mitochondrial ATP production maintains the global ATP pool, indirectly fueling the lysosomal V-ATPase proton pump to maintain acidic luminal pH essential for hydrolase activity and autophagy-mediated degradation^37,100,101^. Reciprocally, lysosomal nutrient-sensing pathways involving V-ATPase, such as V-ATPase–mTORC1 signaling, regulate mitochondrial functions and biogenesis^38^. In addition, mitochondria and lysosomes interact together via metabolite-mediated signaling mechanisms. For instance, mitochondrial reactive oxygen species (mtROS) can oxidatively activate lysosomal TRPML1 channels, leading to calcineurin-dependent dephosphorylation and nuclear translocation of TFEB, which in turn promotes lysosomal biogenesis and autophagy in response to mitochondrial stress^102^. Similarly, mitochondrial dysfunction can lead to NAD^+^ depletion and reduced glycolytic ATP generation near the lysosomal membrane, impairing local V-ATPase function and lysosomal acidification^103^. This bidirectional communication facilitates efficient energy metabolism, organelle quality control and cellular survival.

Dysfunctional mitochondria undergo lysosome-mediated mitophagy, whereas lysosomal pH or signaling alterations modulate mitochondrial respiratory function and longevity^104^. Mechanistically, V-ATPase acts as a signaling interface; for example, mitochondrial proteotoxic stress activates mTORC1 through lysosomal V-ATPase, inducing ATF4 phosphorylation and mitochondrial unfolded protein response (UPRmt) and thereby maintaining cellular redox balance and viability^38^. Conversely, compromised mitochondrial respiration reduces lysosomal V-ATPase activity and AMPK signaling, elevating lysosomal pH, impairing degradation capacity and accumulating damaged mitochondria^101^. Thus, mitochondria and lysosomes dynamically orchestrate metabolism, autophagy and cell fate via the integrated feedback loops and signaling pathways under physiological conditions.

### MLCs in metabolic and quality control

MLCs are dynamic, membrane fusion-independent junctions facilitating direct communication between mitochondria and lysosomes, and crucial for metabolic regulation and organelle quality control under basal conditions^105^ (Fig. 3). At the MLCs, ions and metabolites are exchanged bidirectionally. For instance, lysosomal Ca^2+^ release through TRPML1 channels increases cytosolic Ca²⁺, which subsequently enters mitochondria via VDAC1 channels, elevating mitochondrial matrix Ca²⁺ and enhancing mitochondrial bioenergetics^106^. Pharmacological TRPML1 activation selectively boosts mitochondrial Ca^2+^ uptake at these contacts, whereas pore-inactive TRPML1 mutants impair this transfer^39,106^. MLCs also regulate organelle dynamics through the lysosomal GTPase Rab7, which, in its GTP-bound form, mediates lysosome tethering to mitochondria, and mitochondrial Fis1-driven recruitment of TBC1D15 subsequently hydrolyzes Rab7-GTP, disassembling contacts and facilitating mitochondrial fission^107^. In addition, MLCs coordinate mitophagy via mitochondrial receptors, such as FUNDC1, which are associated with mitochondrial fission (DRP1) and fusion (OPA1) regulators to couple mitochondrial fragmentation with autophagic turnover^108^. These mitochondria–lysosome interactions thus ensure effective nutrient signaling, Ca^2+^ homeostasis and prompt removal of dysfunctional mitochondria. Therefore, disruptions in these contacts are increasingly implicated in neurodegenerative diseases.

### Role of V-ATPase in mitochondrial function

V-ATPase on lysosomal membranes maintains an acidic lysosomal lumen, essential for the exchange of metabolic and signaling molecules at MLCs (Fig. 3). Dysfunction of V-ATPase, leading to elevated lysosomal pH, impairs mitophagy, disrupts clearance of damaged mitochondria and diminishes cellular mitochondrial quality control^109^. This defect results in the accumulation of damaged mitochondria, characterized by compromised mitochondrial membrane potential, reduced ATP production and increased reactive oxygen species (ROS)^36^. Studies in yeast show a direct link between elevated vacuolar pH and mitochondrial dysfunction, demonstrating its role in cellular metabolic homeostasis^110^. Notably, these mitochondrial defects result primarily from the impaired amino acid storage capacity associated with vacuolar lumen pH rather than from defects in vacuolar protein degradation. Similarly, in mammalian cells, the impaired V-ATPase function at the mitochondria–lysosome contacts negatively affects the exchange of crucial signaling molecules, including Ca^2+^ ions, thus undermining mitochondrial functionality and longevity^36,111^. Therefore, proper maintenance of V-ATPase activity is critical for mitochondrial quality control and functional integrity.

### Mitochondria–lysosome crosstalk in neurodegenerative diseases

Recent studies underscore mitochondria–lysosome interactions as a central to cellular homeostasis, with disruption implicated commonly across neurodegenerative diseases (Fig. 3). Simultaneous impairment of mitochondrial and lysosomal functions creates a pathological feedback loop. In AD models, Aβ oligomers aberrantly activate mTORC1 at the plasma membrane while inhibiting its activation at the lysosome, suppressing mitochondrial activation despite nutrient signals^112^. In addition, Aβ and tau disrupt mitophagy to promote abnormal mitochondrial accumulation^113^. Lysosomal dysfunction resulting from ApoE4 expression, a genetic risk factor in AD, impairs mitophagy in human astrocytes, leading to the accumulation of dysfunctional mitochondria. This lysosomal impairment is linked to altered mitochondrial dynamics, reduced oxidative phosphorylation and bioenergetic stress, thereby exacerbating mitochondrial dysfunction under pathological conditions^48^. Tau aggregates particularly increase mitochondrial membrane potential, directly inhibiting Parkin-mediated mitophagy and leading to toxic accumulation of damaged mitochondria in neurons^114^.

In PD, mitochondria–lysosome dysfunction manifests through impaired mitophagy and aberrant MLCs in neuronal cells. Defects in the PINK1–Parkin pathway, essential for selective mitochondrial clearance, contribute centrally to PD pathogenesis through cumulative mitochondrial dysfunction^115^. This mitochondrial impairment secondarily triggers lysosomal dysfunction, reinforcing a pathological cycle between these organelles^116^. GBA1 mutations in PD models abnormally increase MLCs, which disrupts mitochondrial function and positioning, thus impairing cellular homeostasis^117^. In addition, PINK1 facilitates intercellular pathological signaling via extracellular vesicles containing mitochondrial DNA^118^. In glial cells, particularly under PD stress conditions, mitochondrial dysfunction has been shown to perturb mitochondria–lysosome communication, leading to compromised lysosomal degradative function and aberrant exosome release, which may propagate pathological proteins and amplify neuroinflammatory responses^45,119^. Gaucher disease models further emphasize the connection between GBA1 deficiency and concurrent mitochondrial–lysosomal quality control defects, reinforcing the disease’s association with PD^120^.

In HD, excessive mitochondrial fragmentation mediated by Drp1 and Fis1 directly impair lysosomal function. The mHtt protein promotes mitochondrial fragmentation, overwhelming lysosomal processing capacity, compromising degradation and reducing neuronal viability^121^. In lysosomal storage disorders, pathological suppression of mitochondrial biogenesis occurs. Disorders characterized by lysosomal lipid accumulation, such as Niemann–Pick disease or acid sphingomyelinase deficiency, activate transcription factors (KLF2 and ETV1) that inhibit PGC-1α, the master regulator of mitochondrial biogenesis. This limits mitochondrial abundance and reduces respiratory capacity, severely impairing neuronal energy metabolism^122^.

Collectively, these diseases share a common pathological cycle involving mitochondrial and lysosomal dysfunction. ROS generated from impaired mitochondria disrupt lysosomal acidification, diminish autophagic efficiency and exacerbate mitochondrial accumulation^123^. Notably, compromised lysosomal acidification via V-ATPase dysfunction emerges as a central pathological factor, linking impaired mitochondrial quality control to altered mitochondria–lysosome interactions in aging and neurodegeneration^39,40^. Therefore, restoring and maintaining the mitochondria–lysosome interactions are increasingly recognized as pivotal therapeutics for neurodegenerative diseases.

## Lysosomal acidification as a therapeutic target for endomembrane system integrity

Lysosomal acidification has emerged as an attractive therapeutic target due to its central role in maintaining cellular homeostasis and regulating interactions among organelles within the endomembrane system, such as the ER, Golgi apparatus and mitochondria^111^. Given that lysosomal dysfunction substantially disrupts the interorganelle communication and cellular quality control essential in neurodegenerative diseases, targeted modulation of V-ATPase activity offers promising therapeutic potential^3,10^. Recent studies underscore that restoring lysosomal function can broadly stabilize organelle dynamics and cellular homeostasis, highlighting its therapeutic potential.

### Restoring lysosomal pH

Recent therapeutic strategies target V-ATPase to normalize lysosomal pH and enhance protein degradation. A recent study reported that the small molecule C381 directly stimulates V-ATPase activity, restores lysosomal acidity and improves proteolytic efficiency, thereby reducing inflammation and neuropathology in neurodegenerative models^124^. Indeed, C381 administration protected dopaminergic neurons and improved cognitive function in PD models. Another study demonstrated that EN6, targeting the V1A subunit of V-ATPase, enhances lysosomal acidification and inhibits mTORC1 signaling by disrupting V-ATPase–Rag GTPase interaction, notably facilitating the clearance of pathological TDP-43 aggregates in frontotemporal dementia and ALS models^125^. In addition, decrease of V-ATPase subunit expression was identified as a pathogenic event in AD, and histone deacetylase inhibitor NCH-51 was shown to restore *ATP6V1A* expression, consequently normalizing lysosomal function and neuronal viability^126^. Therefore, strategies aiming at boosting V-ATPase activity or increasing its subunit expression represent promising therapeutic avenues for the treatment of neurodegenerative diseases. Nanoparticle-based therapeutic strategies are also promising. For instance, PLGA-derived acidic nanoparticles (aNPs) were utilized to restore lysosomal acidity in cellular models impaired by ATP13A2 mutations or GBA deficiency. In PD mouse models, aNP administration led to lysosomal reacidification, reduced dopaminergic neuron degeneration and mitochondrial dysfunction, and improved neuronal survival^126^.

### Restoration of organelle network integrity

As discussed earlier, restoration of organelle network integrity by modulating V-ATPase activity can alleviate neurodegenerative pathology. The β2-adrenergic agonists, such as isoproterenol, were utilized in PS1-deficient cells and fibroblasts derived from patients with familial AD to normalize lysosomal pH, restoring lysosomal proteolytic function, improving intracellular Ca^2+^ homeostasis and normalizing autophagic flux. These agonists also correct the impaired ER-to-lysosome ion transport involving ClC-7 and alleviate autophagy and lysosomal dysfunction characteristic of AD pathology^127^. Rab GTPase, highlighted as a master regulator in ER–Golgi–endosome–lysosome trafficking, has been targeted therapeutically in AD. Modulating Rab activity improved protein trafficking and reduced amyloid accumulation, validating organelle network restoration as an effective therapeutic strategy for neurodegenerative conditions^128^. Chemical chaperones targeting both ER and lysosomes prevented neurodegeneration in C9orf72 ALS models by enhancing protein folding, thereby reducing toxic protein accumulation and preserving organelle function^129^. In addition, DDPU, a protopanaxadiol derivative from ginseng, reduced ER stress and enhanced autophagy in APP/PS1 AD mice by inhibiting PI3K/mTOR signaling, promoting lysosomal clearance of Aβ and restoring ER–lysosomal function^130^. Furthermore, Parkin-deficient PD neurons exhibit decreased mitochondria–lysosome contacts, leading to excessive amino acid accumulation in lysosomes and mitochondrial deficits. By inhibiting TBC1D15, a regulator of Rab7 activity, mitochondria–lysosome contacts were also restored, reinstating amino acid homeostasis and alleviating metabolic dysfunction^131^.

## Future directions and concluding remarks

A fundamental therapeutic approach in neurodegenerative diseases necessitates strategies beyond traditional single-target therapies, instead requiring integrated restoration of interorganelle networks involving the ER, Golgi apparatus, mitochondria and lysosomes. Lysosomal acidification defects trigger ER stress, Golgi fragmentation and mitochondrial dysfunction, fostering pathological synergy among these organelles. Thus, combinatorial therapy or polypharmacology, aiming at restoring interdependent organelle functions, presents a promising avenue. The rationale for multitarget interventions is supported by evidence that damage to individual organelles disrupts overall cellular homeostasis. Advances in nanotechnology further enable precise and tailored drug delivery systems, increasing clinical feasibility. For instance, combining lysosomal aNP with mitochondrial activators selectively targeting neurons could optimize therapeutic synergy while minimizing adverse effects.

Critically, lysosomal acidification mediated by V-ATPase remains a key therapeutic opportunity due to its fundamental role in maintaining neuronal proteostasis and preventing harmful cascades of organelle dysfunction. To fully harness this therapeutic potential, future research should prioritize developing sensitive and reliable methods for monitoring lysosomal pH at early disease stages, enabling proactive rather than reactive clinical interventions. Furthermore, refining multitarget therapeutic strategies and advancing sophisticated drug delivery systems or gene therapy techniques to precisely modulate organelle functions represent critical next steps. Ultimately, integrating these proactive therapeutic approaches holds substantial promise for developing truly transformative treatments. Such comprehensive approaches, focusing on early intervention and precise modulation of interorganelle interactions, could considerably improve therapeutic outcomes for AD, PD, ALS and other neurodegenerative disorders.
